# Risk of Mortality (including Sudden Cardiac Death) and Major Cardiovascular Events in Users of Olanzapine and Other Antipsychotics: A Study with the General Practice Research Database

**DOI:** 10.1155/2013/647476

**Published:** 2013-12-14

**Authors:** Meghan E. Jones, Giedra Campbell, Deven Patel, Elizabeth Brunner, Chetan C. Shatapathy, Tarita Murray-Thomas, Tjeerd P. van Staa, Stephen Motsko

**Affiliations:** ^1^Lilly Corporate Center, Eli Lilly and Company, Indianapolis, IN 46285, USA; ^2^Clinical Practice Research Datalink, Medicines and Healthcare Products Regulatory Agency, London SW1W 9SZ, UK; ^3^Utrecht Institute for Pharmaceutical Sciences, Utrecht University, Utrecht, The Netherlands; ^4^London School of Hygiene & Tropical Medicine, London WC1E 7HT, UK

## Abstract

*Objective*. Assess risk of cardiac events and mortality among users of olanzapine and other antipsychotics relative to nonusers. *Methods*. The General Practice Research Database was used to identify cohorts of antipsychotic users and nonusers with psychiatric illness. Outcomes included cardiac mortality, sudden cardiac death (SCD), all-cause mortality (excluding suicide), coronary heart disease (CHD), and ventricular arrhythmias (VA). *Results*. 183,392 antipsychotic users (including 20,954 olanzapine users) and 193,920 psychiatric nonusers were identified. There was a significantly higher rate of cardiac mortality (adjusted RR [aRR]: 1.53, CI, 1.12–2.09) in olanzapine users relative to psychiatric nonusers, consistent with findings for both atypical and typical antipsychotics. Relative to psychiatric nonusers, no increased risk of all-cause mortality was observed among olanzapine users (aRR: 1.04, CI, 0.93–1.17), but elevated all-cause mortality risk was observed when compared to all antipsychotic users (aRR: 1.75, CI, 1.64–1.87). There was no increased risk of CHD or VA among olanzapine users relative to psychiatric nonusers, consistent with findings for atypical but not typical antipsychotics. SCD cases were uncommon. *Conclusions*. Use of antipsychotic agents was associated with increased risk of all-cause and cardiac mortality. Patients treated with olanzapine were found to be at increased risk of cardiac mortality versus psychiatric nonusers.

## 1. Introduction

It is generally accepted that that there is an increased risk of mortality (including sudden cardiac death (SCD)) and major cardiac events during treatment with some antipsychotics in patients with schizophrenia and other mental illnesses [1–5]. Several large observational studies have been conducted in recent years to assess the extent of increased risk with these outcomes in antipsychotic users and to differentiate risk with typical and atypical antipsychotics. In a retrospective cohort study of Medicaid enrollees in Tennessee, Ray et al. [2] found that current users of typical and atypical antipsychotics had a similar, dose-related increased risk of sudden cardiac death. The primary objective of the present retrospective observational study was to assess the risks of sudden cardiac death and cardiac mortality among users of the antipsychotic product olanzapine. The risk associated with olanzapine prescribing was assessed relative to psychiatric patients who did not use antipsychotic medications (nonusers) and among groups of antipsychotic medication users (atypical, typical, and any), as well as the risks among users of individual antipsychotic agents. Secondary outcomes included all-cause mortality (excluding suicide) and several nonfatal cardiovascular outcomes. Results evaluating risks for antipsychotic users in general, relative to nonusers with psychiatric illness and to the general population, are being published elsewhere. This paper focuses on the risk associated with olanzapine treatment relative to a nonuser psychiatric population, presented in the context of risks associated with other types of antipsychotics and individual antipsychotic agents.

## 2. Methods

### 2.1. Study Population

The General Practice Research Database (GPRD), now administered by the Clinical Practice Research Datalink, was used to identify populations for the study. GPRD comprises anonymised computerised medical records from primary care for about 8% of the UK population. At the time of the study, about 40% of GPRD practices also participated in anonymous and patient-level linkages to the national registry of hospital admission (Hospital Episode Statistics (HES)) and death certificates (as collected by the Office of National Statistics (ONS)). The base population was comprised of all acceptable patients registered in GPRD from January 1995 to January 2011. The study populations were limited to acceptable patients from up-to-standard practices to ensure the quality of the data. Patient acceptability is based on eleven assessment criteria defined by the GPRD to ensure that registered patients meet basic research quality standards. Up-to-standard practices are general surgery practices that meet a practice-based quality marker indicating when a practice has reached an acceptable level of data entry quality and thus are considered to have continuous high quality data.

Patients were also required to be registered in GPRD for at least one year prior to the index date (cohort entry date) to ensure adequate assessment of medical history and prior drug usage. Patients with a history of life-threatening ventricular tachyarrhythmia, cardioversion, aborted cardiac arrest, or implantation of a cardiac defibrillator in the baseline period were excluded. Patients with a congenital conduction disorder or advanced cardiomyopathy (hypertrophic or dilated) prior to the index date or at any time during follow-up were also excluded. HES data were available from April 1997 and death certificates from January 2001. The data from HES, death certificates, and GPRD were recorded and collected independently from each other.

One cohort of current antipsychotic users was classified at the index date (first date of antipsychotic prescribing in the study period) into prevalent and incident antipsychotic users: incident users were those with a first-ever prescription; prevalent users were those who received an antipsychotic prescription before index. For some analyses, this cohort was further subdivided by type of antipsychotic (typical versus atypical, or by specific antipsychotic).

A cohort of patients with a diagnosis of schizophrenia, bipolar disorder/other mood disorders, major depression, or dementia but without a history of use of antipsychotics, referred to as psychiatric nonusers, was also identified. For this cohort, the index date was defined as the date of the first record of the psychiatric disorder (i.e., both incident and prevalent cases were included). Patients were censored at the earliest of their deregistration date at the practice, the date of their first antipsychotic prescription after their diagnosis date (if they remained registered at the practice), the last collection date of the practice, or the end of the study period. This cohort was not matched to the cohort of antipsychotic users; rather, baseline differences were adjusted for using multivariate analysis.

For both cohorts, patients were followed from the index date up to the occurrence of the outcome of interest or the end of data collection (i.e., last GPRD data collection, transfer out of the practice, or date of death, whichever date came first). For antipsychotic users, the follow-up was restricted to “current exposure,” defined as the period from the initial antipsychotic prescription up to one month after the expected end of treatment. The defined daily dose (DDD) for prescriptions was calculated based on the prescribed daily dose and strength per tablet. These DDDs were then classified as low, medium, or high dose using the dose thresholds for antipsychotics defined in terms of chlorpromazine equivalents [6]. Low daily dose was defined as <200 mg, medium as 200–399 mg, and high as ≥400 mg chlorpromazine equivalents per day.

Duration of use was calculated from cohort entry date to the expected end of the last prescription occurring prior to their censor date. Duration of use was defined as the prescription quantity divided by daily dose. Where the estimated duration was missing or corresponded to approximately the 1st and 99th percentiles of the distribution of all estimated prescription durations, estimated durations were imputed using the median for that prescription. Patients whose duration of use could not be estimated in this way were assigned the median pertaining to the subtype of the antipsychotic substance instead. About 13% of all prescriptions were imputed. The cumulative treatment duration at the end of follow-up for all exposed patients was calculated as tertiles which corresponded broadly to <1 year, 1–3 years, or >3 years of use.

### 2.2. Ascertainment of Outcomes of Interest

The outcomes of interest included cardiac mortality, three definitions for sudden cardiac death (SCD), all-cause mortality excluding suicide (referred to as all-cause mortality from here on out), coronary heart disease (CHD), and life-threatening ventricular arrhythmias (VA). Outcomes were identified using GPRD Read codes, ICD10 codes from ONS death certificate data and records from HES (Table 1).

When querying multiple sources for the occurrence of an outcome, the following hierarchy was used, with earlier-named sources taking precedence over the later-named sources when more than one was available for any given patient: death certificate (both the recorded date and cause of death), followed by GPRD free text (date of death taken to be the earlier of the recorded GPRD date of death or free text date), followed by GPRD Read code (date of death taken to be the earlier of the recorded GPRD date of death or the date of Read code). Where both a Read code and a free text term were recorded, the earlier of the two events was considered as the date of the event. For example, if under the sudden cardiac death primary definition a patient would be considered a case based on the GPRD Read codes but not on their cause of death, then the patient was not recorded as a case. However, if the definition was based on GPRD Read codes the patient would be considered a case supporting evidence from free text was not also needed. A conservative approach in identifying the SCD cases was used, counting them as SCD cases unless the free text clearly specified another cause of death. In addition, GPRD free text information was used to improve the odds of accurately identifying SCD cases. For relevant cases from practices shown to use an above-median amount of free text, 2 independent experts reviewed free text surrounding search string terms suggestive of sudden cardiac death (Table 1) and identified events appropriate for inclusion.

### 2.3. Statistical Analyses

Poisson regression analysis was used to examine the rates of each outcome and produce corresponding age- and sex-adjusted (RRs) and fully adjusted relative ratios (aRRs) and 95% confidence intervals (CI) for each comparison. Adjusted models were fitted using automated backward elimination with stepwise regression. To ensure that the resulting models were not overparameterised, the number of variables to be included in the model was selected using the rule of thumb of five outcome events per parameter [7, 8]. Goodness-of-fit was assessed by examining the associated *P* values of the covariates such that a covariate was included in the model only if the associated *P* value was <0.05, while any covariates in the model with *P* values ≥ 0.10 were excluded. The following patient characteristics were considered as potential confounders in regression models: age, sex, socioeconomic status (Index of Multiple Deprivation calculated at the postcode of the patient's residence), smoking history, alcohol use, and body mass index (BMI). Age, sex, and treatment were included in the model a priori. BMI was calculated using the most recent BMI measurement available before the index date. Nonsmokers were classified as patients with a history of nonsmoking only, documented in their record. Current smoking was based on the most recent smoking record of the patient documented before the index date. Regression models included indicator variables for missing values for BMI and smoking. Other confounding factors included time since start of GPRD data collection (in tertiles); duration of psychiatric disease; history of cardiovascular disease; alcohol or drug abuse; diabetes mellitus; history of suicide attempt; prior hospital admission for psychiatric disease; and prescribing in the previous 3 months of statins or fibrates, antihypertensive drugs, warfarin, antiplatelets, nitrates, lithium, antiepileptics, antidepressants, and anxiolytics. To examine changes in risk over time, patient follow-up (from start of medication until end of data collection) was subdivided into 100 periods of equal length and incidence rates (hazard rates) were estimated for each period. These rates were smoothed using the methods proposed by Ramlau-Hansen [9]. Analyses were conducted using STATA 11.

## 3. Results

The study identified 183,392 antipsychotic users (typical or atypical), including 20,954 olanzapine users (11.4%), and 193,920 patients with psychiatric illness who were not users of typical or atypical antipsychotic drugs (psychiatric nonusers) (Table 2). The average duration of follow-up time was 4.0 years (SD 4.0) for antipsychotic users, 3.6 years (SD 2.86) for olanzapine users, and 4.1 years (SD 3.5) for patients with psychiatric illness. The olanzapine and psychiatric nonuser populations, which were not matched to minimize heterogeneity, differed in some respects. Whether these differences are statistically or clinically significant is unknown.

This study assessed all-cause and cardiac mortality, SCD, CHD and ventricular arrhythmias among patients currently taking antipsychotic agents relative to nonusers of antipsychotic drugs.

### 3.1. All-Cause and Cardiac Mortality

Significantly higher risk for cardiac mortality was observed in patients currently treated with olanzapine relative to nonusers with psychiatric illness (aRR: 1.53, 95% CI, 1.12–2.09) (Table 3). This result is consistent with cardiac mortality risk findings for antipsychotic users in general (aRR: 1.72, 95%CI, 1.42–2.07). An assessment of all-cause mortality found no increased risk for patients treated with olanzapine relative to nonusers with psychiatric illness. In contrast, there was a significantly elevated risk for all-cause mortality for antipsychotic users as a group and other specific agents assessed (except perphenazine) relative to nonusers with psychiatric illness (Table 4).

### 3.2. Sudden Cardiac Death

Due to small event counts, it was not possible to do a comparative assessment of SCD risk for patients treated with olanzapine (using any of the 3 definitions). Increased risks of SCD (secondary definition (aRR: 2.15, 95% CI, 1.64–2.81) and tertiary definitions (aRR: 1.79, 95% CI, 1.42–2.27)) were observed for patients currently treated with antipsychotics relative to nonusers with psychiatric illness (Table 3). As with the olanzapine cohort, there were insufficient event counts to assess the primary definition for typical and atypical users individually. Using the secondary and tertiary definitions, current users of typical antipsychotics had a higher magnitude of SCD risk than users of atypical antipsychotics.

### 3.3. Coronary Heart Disease and Ventricular Arrhythmias

There were no increased risks of CHD and ventricular arrhythmias for patients currently treated with olanzapine relative to nonusers with psychiatric illness (Table 3). For both outcomes, this is consistent with findings for users of other atypical antipsychotics. In the case of CHD, there was a statistically significant increased risk among typical antipsychotic users relative to nonusers.

### 3.4. Outcomes Stratified by Age

**Figure 1:**
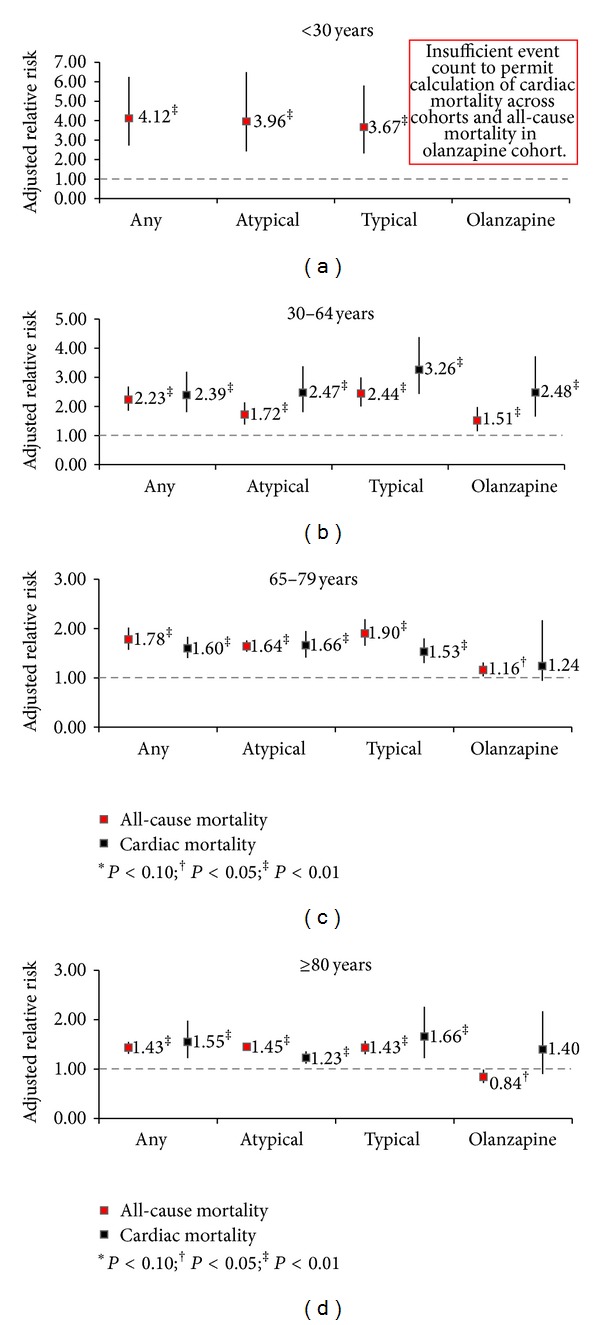
Relative risks of all-cause mortality (excluding suicide) and cardiac mortality for current users of various types of antipsychotics versus nonuser psychiatric controls stratified by age.

Olanzapine patients aged 30 to 64 had statistically significant increased risk of all-cause mortality compared to nonusers with psychiatric illness from the same age group (aRR: 1.51, 95% CI, 1.15–1.98) (Figure 1). Olanzapine patients aged 65 to 79 had a small but significantly increased all-cause mortality risk relative to nonusers with psychiatric illness in this age group (aRR: 1.16, 95% CI, 1.03–1.31). Olanzapine patients 80 and above had statistically significant lower all-cause mortality risk relative to nonusers aged 80 and above (aRR: 0.84, 95% CI, 0.72–0.99). There were not enough mortality events among olanzapine patients under the age of 30 to enable comparisons. This pattern of findings, with higher relative risks from 30 to 64 year old compared to the older age strata, was similar for the cardiac mortality and CHD outcomes for olanzapine (data for CHD not shown) and was also observed for all-cause and cardiac mortality risk, for users of atypical agents, typical agents, and antipsychotics in general. For both SCD and ventricular arrhythmias, there were insufficient event counts to calculate aRR by olanzapine age strata (data not shown).

### 3.5. Outcomes Stratified by Dose

Olanzapine users taking medium and high doses had a significantly increased risk of cardiac mortality relative to a nonuser psychiatric population (Table 5). Significantly increased risk of cardiac mortality was also observed at all dose levels for patients taking any atypical or any typical antipsychotic agent.

Due to low counts of SCD among users of olanzapine, risk for SCD could not be calculated by dose. Significantly increased risks of SCD with increased dose were observed for users of typical antipsychotics or for any antipsychotic; however, there was no apparent pattern for users of atypical antipsychotics, for whom the highest increased risk for SCD was seen with low doses, followed by high and then medium doses. It should be noted, however, that even in the larger overall antipsychotic user groups, there were small event counts (<15) in the medium- and high-dose strata, limiting the interpretation of the results.

For all-cause mortality, there was no evidence of increasing risk with increasing dose for patients treated with olanzapine or for patients treated with atypical antipsychotics in general, relative to nonuser psychiatric patients. However, patients treated with typical antipsychotics had increasing risk with increasing the dose from low (aRR of 1.76) to medium (aRR of 3.74).

For CHD, there was no evidence of increasing risk with increasing dose for patients treated with olanzapine, atypical antipsychotics, or typical antipsychotics. The only significant result was risk for patients receiving low-dose typical antipsychotics.

For ventricular arrhythmia, there was no evidence of increasing risk with increasing dose for patients treated with olanzapine or for patients treated with atypical antipsychotics in general, relative to nonuser psychiatric patients. This is in contrast to patients using typical antipsychotics, with markedly higher risk ratios for ventricular arrhythmias among patients taking medium (aRR of 2.23) and high doses (aRR of 2.11) versus low doses (aRR of 1.13), although only the risk associated with the medium dose was statistically significant.

### 3.6. Outcomes Stratified by Duration of Treatment

Table 6 summarizes results related to cardiac mortality and all-cause mortality by duration of treatment (<1 year, 1–3 years, and longer than 3 years) relative to nonusers with psychiatric illness. Among patients currently taking olanzapine, the risk of all-cause and cardiac mortality was lowest for those who had been treated for >3 years. The risk of cardiac mortality was significantly elevated for patients receiving olanzapine for <1 or 1 to 3 years, but the risk of all-cause mortality was only significantly elevated for patients receiving olanzapine for <1 year. Among the larger group of all antipsychotic users, the overall cardiac mortality risk was significantly elevated for all 3 duration strata; however, the risk decreased as duration of use increased, which is similar to the trend seen with users of olanzapine. For CHD and ventricular arrhythmia, very small differences were seen between the duration of treatment strata for olanzapine and for overall typical and atypical antipsychotic drug users (data not shown). Again, due to small counts, analyses did not produce reliable results for comparison of strata by duration of treatment for any of the three definitions of SCD for current users of olanzapine.

### 3.7. Comparison to Unexposed, Nonpsychiatric Population

While previous results describe findings within the olanzapine cohort relative to nonusers with psychiatric illness, a comparison with the general population (i.e., age- and gender- matched patients without schizophrenia, major depression, etc.) was also conducted. This cohort comprised 544,726 individuals. Despite differences in baseline psychiatric illness, risks for all-cause cardiac mortality, SCD, CHD, and ventricular arrhythmia outcomes for current users of olanzapine relative to the general population were similar to risks for these outcomes relative to nonusers with psychiatric illness. However, there was a difference found in the all-cause mortality comparison for olanzapine: risk for olanzapine users was higher relative to the general population (aRR: 1.87, 95% CI, 1.69–2.08) than the risk relative to nonusers with psychiatric illness (aRR: 1.04, 95% CI, 0.93–1.17).

## 4. Discussion

Patients treated with olanzapine had a significantly increased risk of cardiac mortality relative to psychiatric patients not using antipsychotics. The number of SCD events in olanzapine users was too low to calculate risk ratios. Patients treated with olanzapine were not shown to have higher risk for CHD and ventricular arrhythmia relative to nonusers, such that it is unclear what led to the increased risk of cardiovascular mortality overall. Although we found that the rates of CHD between the exposed cohort compared to psychiatric nonusers were similar, this observation may be partly explained by the very narrow definition used to identify CHD in the study. Increased risk for adverse cardiovascular outcomes among patients with schizophrenia has previously been reported [11, 12], and the olanzapine-treated population had a markedly higher proportion of patients with schizophrenia than did the nonuser cohort; however, this does not explain why risk for cardiac mortality was increased, whereas risk for the secondary outcomes of CHD and ventricular arrhythmias was not increased. Furthermore, patients treated with olanzapine were not found to be at significantly increased risk of all-cause mortality relative to psychiatric nonusers.

Olanzapine patients, age 30–64, were found to be at significantly higher risk for several of the outcomes (all-cause and cardiac mortality) than patients in older age strata; there were too few patients/events in the <30 age group for adequate assessment of younger patients. Generally, the lowest risk was for patients ≥80 years old. The lower magnitude of risk in people in the oldest age stratum may represent a healthy survivor effect. No patterns were observed that suggested that increased risk was associated with increased duration of olanzapine use, which contradicts the hypothesis that increased risk of adverse cardiac outcomes associated with olanzapine use may be via a metabolic mechanism of action. However, it is possible that negative cardiac outcomes might have been avoided if, for example, patients with adverse metabolic changes early in treatment discontinued drug earlier than patients without such changes.

In this study, use of antipsychotic agents in general, whether typical or atypical, was associated with increased risk of all-cause mortality and of cardiac mortality. In addition, users of antipsychotic agents had an increased risk of SCD.

We compared the results from our study to those in other available studies of the cardiovascular risk with antipsychotic use. Ray et al. [2] calculated the adjusted incidence of SCD among current users of atypical antipsychotic drugs in a retrospective cohort study of Medicaid enrollees in Tennessee. This study reported an elevated risk of SCD (aRR 2.26; 95% CI, 1.88–2.72) among atypical antipsychotic users compared to a nonuser psychiatric population. The present study results show a similar finding when using the secondary definition of SCD (aRR 1.79; 95% CI, 1.33–2.42; note that the secondary definition of SCD used in the current study is the one most consistent with that used by Ray et al.). In the current study, the risk for SCD relative to nonusers was over twice as high for typical antipsychotics as for atypical antipsychotics (aRR 3.99, 95% CI, 1.29–12.40 versus 1.79, 95% CI, 1.33–2.42); this finding contrasts with Ray's finding of more similar rate ratios for SCD among current users of typical and atypical antipsychotics (aRR 1.99, 95% CI, 1.68–2.34 versus 2.26, 95% CI, 1.88–2.72). Ray et al. also reported an increasing risk of SCD with the increasing atypical antipsychotic dose, a finding which was not borne out in the present study, although we did find increasing risk with increased doses of typical antipsychotics. One noteworthy difference between Ray et al. and the present study that could have affected outcomes is that Ray's cohort of antipsychotic users had a mean age of 46, while the cohort of antipsychotic users in our study had a mean age of 60. This discrepancy is due to the nature of the studied databases. Whereas patients of all ages are included in the GPRD database, Ray et al. studied patients enrolled in Medicaid, who leave Medicaid at the age of 65 to enroll in Medicare.

One limitation of our study with respect to assessment of risk for SCD is that accurately identifying cases of SCD from electronic medical record codes are inherently challenging. In an effort to overcome the known limitations [13], we also used free text searches. Cases were counted as SCD based on coding algorithms unless the available free text clearly specified another cause of death. However, this approach was still limited, as many cases had little or no free text, and often the free text said nothing more than “sudden death” or “died suddenly.” When more information was available, it was often determined that the patient did not experience SCD (e.g., deaths due to motor vehicle accident, drug overdose). These factors suggest a relatively high number of false positives. Given these concerns, along with the relatively small number of cases overall, data regarding SCD must be interpreted cautiously.

Enger et al. [5] compared the risk of all-cause mortality (excluding suicide), cardiovascular mortality, acute myocardial infarction (MI), and arrhythmias in people with schizophrenia who use antipsychotic medications to risks in individuals without schizophrenia in a large managed care organization. Our analysis assessing risk of all-cause mortality for users of any antipsychotic relative to psychiatric nonusers (1.75 [95% CI, 1.64–1.87]) is consistent with that in Enger et al. (2.18 [95% CI, 1.14–4.18]). Among atypical antipsychotic users, the aRR for all-cause mortality in the current study was 1.52 (95% CI, 1.40–1.64) versus Enger's aRR of 1.47 (95% CI, 0.14–15.05); these values are similar, but the wide confidence intervals in the Enger study should be noted. Adjusted RRs for ventricular arrhythmias among users of atypical antipsychotics are similar in both this study (1.16 (95% CI, 1.02–1.31)) and that of Enger et al. (1.01 (95% CI, 0.09–11.28)), although the current study found a statistically significant increased risk. Adjusted RRs for CHD among users of atypical antipsychotics were nonsignificant in both the current study (0.98 (95% CI, 0.76–1.26)) and in that of Enger et al. (1.66 (95% CI, 0.19–14.82)). Of note, the Enger study was composed of patients diagnosed only with schizophrenia, whereas the present study population included a broader psychiatric population with severe mental illness.

Osborn et al. [14] carried out a historical cohort study comparing mortality from CHD, cancer, and stroke between a cohort of patients with severe mental illness (SMI), including schizophrenia and bipolar disorder, and a cohort without SMI, using GPRD. The risk of death was stratified by age, use, and nonuse of antipsychotic agents (typical and atypical combined), as well as by dose of antipsychotic agents. The study found a higher relative rate of CHD-related death among the 18–49 year age strata as compared to those 75+ (hazard rate 2.88 versus 1.04). Although there were insufficient counts in the age stratum younger than 30 in the current study, a similar trend of increased risk for younger versus older patients was seen for both the CHD-related deaths and all-cause mortality in the current study. Neither Osborne et al. nor the current study showed a trend for increasing CHD-related death as dose increased.

An 11-year population-based cohort study with follow-up of mortality in schizophrenia patients [15] that is not directly comparable to our study showed that long-term cumulative exposure (7–11 years) to any antipsychotic treatment was associated with lower all-cause mortality than was no antipsychotic use (aRR 0.8 [95% CI: 0.77–0.84]). There were no significant differences in risk of death from ischaemic heart disease among all the antipsychotics studied (risperidone, thioridazine, quetiapine, haloperidol, perphenazine, clozapine, and olanzapine). Olanzapine was not associated with an increase in risk of mortality from ischaemic heart disease when compared with perphenazine (aRR 0.88 [95% CI: 0.63–1.21]) and was associated with a lower total mortality and deaths due to ischaemic heart diseases than the other antipsychotics [15].

More recently, Leonard et al. [16] calculated adjusted hazard ratios (HR) of SCD or VA (together) and all-cause death among users of antipsychotic drugs in a retrospective cohort of Medicaid enrollees in California, Florida, New York, Ohio, and Pennsylvania. Compared to olanzapine as the referent, adjusted HRs for SCD/VA were 2.06 (95% CI, 1.20–3.53) for chlorpromazine, 1.72 (1.28–2.31) for haloperidol, 0.63 (0.34–1.16) for perphenazine, 0.73 (0.57–0.93) for quetiapine, and 1.04 (0.88–1.24) for risperidone. These results are consistent with our study findings suggesting greater cardiac risks for users of typical antipsychotics versus atypical antipsychotics.

Our study did not evaluate mechanisms that might explain increased risk in cardiac mortality among antipsychotic users; however, 2 main mechanisms have been proposed. The first mechanism is blockade of potassium channels and prolongation of ventricular repolarization, which can contribute to potentially fatal ventricular arrhythmias. As such, prolongation of the QTc interval is considered a reasonable marker of increased risk [1, 17]. An increased risk of mortality was observed in patients treated with some antipsychotics which are associated with prolongation of the QT interval and/or adverse changes in metabolic parameters. Antipsychotics vary with respect to the risk of prolonged QT interval, however, and sometimes mortality findings for patients taking medications known to increase QT do not suggest increased risk of acute death; for example, thioridazine, a known inducer of prolonged QT, was not associated with high all-cause mortality in an analysis of current use [15]. Leonard et al. [16] also found that risks for SCD/VA and death did not correlate well with average QT prolongation, suggesting that average QT prolongation may be a poor surrogate of antipsychotic arrhythmogenicity. Previous analyses of clinical trial data have suggested that olanzapine does not increase the risk of QTc prolongations that lead to potentially fatal ventricular arrhythmias [18, 19]; this is supported by findings of the present study that patients treated with olanzapine were not at increased risk of ventricular arrhythmias despite being at increased risk of cardiac mortality in general.

An alternative, or perhaps additional, explanation for increased risk of sudden cardiac death or cardiovascular disease during treatment with antipsychotics could be the increased risk of metabolic changes associated with psychiatric disorders. Patients with schizophrenia or other psychiatric disorders have a higher prevalence of diabetes [14] and/or cardiovascular disease-related morbidity and mortality as compared to the general population [11, 20]. Depression is also a known risk factor for mortality due to coronary heart disease [21]. More than 80% of cases of sudden cardiac death occur in individuals with coronary heart disease [15]. SCD occurs in only 5% to 10% of subjects without a positive history for coronary heart disease or congestive heart failure, and the most common electrophysiological mechanisms leading to SCD are ventricular tachyarrhythmias [22].

As anticipated, there was a significant discrepancy in the distribution of psychiatric illnesses across the user and nonuser groups. The most commonly identified psychiatric diagnosis for the olanzapine cohort was schizophrenia (18%) followed by major depression (16%) and bipolar disorder (10%). Fifty percent of this group had no reported psychiatric diagnosis, although it is likely that many of the olanzapine users without a reported psychiatric diagnosis in fact had schizophrenia. This distribution sharply contrasts with that of the nonuser psychiatric population, where the majority had a diagnosis of major depression (69%) followed by dementia (24%), with only 4% having a diagnosis of schizophrenia and 2%, bipolar disorder. The discrepancy in presence of diagnosis in the database may be partly explained by the fact that more specific mental health diagnoses are likely to be made in secondary care or in the community mental health setting and may not be communicated in a detailed manner back to the general practice; furthermore, there may be stigma associated with psychiatric disorders that may deter documentation. To assess the impact of underlying psychiatric disease, analyses stratified by underlying psychiatric illness were performed. The results showed a 2-fold increased risk of all-cause mortality among patients with schizophrenia (aRR 1.99, 95% CI, 1.50–2.65) as compared to the general population. Major depression had a slight increased risk (aRR 1.05, 95% CI 0.98–1.12) compared to the general population. The other psychiatric disorders analyzed had similar risks with an aRR of 1.45 (95% CI, 1.40–1.50) for dementia and 1.40 (95% CI, 1.01–1.95) for bipolar disorders. It is possible that the imbalance of psychiatric illness between the study cohorts could have contributed to the higher mortality rates among patients taking antipsychotic agents.

This study was based on a large nationally representative community sample of patients diagnosed with psychiatric disease with exposure to antipsychotic agents. The large sample size allowed for systematic evaluation of cardiovascular outcomes of interest (with the exception of SCD due to a limited number of events and validity issues). The sudden cardiac death definition was restricted by the lack of precise timing of the sequence of events that lead to an out of hospital death and the limited clinical detail in the GPRD records. This study attempted to control for differences in patients characteristics; however, it is important to note that some characteristics may not have been adequately controlled for (such as smoking status and physical activity). Another limitation of the study is the inherent difficulty in sorting out the myriad influences on patient outcomes, including the natural course of illness, the positive effects of treating the illness on general health practices, and the potential adverse events during treatment with medication.

We did not assess the validity of the outcomes of interest to this study as the data that are needed to support robust validation, for example, detailed echocardiography report and coroner's reports, are not widely available in secondary sources of data such as GPRD and Hospital Episode Statistic data. Failure to validate outcomes across the different sources of data is another limitation of this analysis of the potential association between cardiac mortality and treatment with olanzapine.

Finally, our observed association between antipsychotic prescribing and cardiac mortality may have been better addressed using propensity score-matched analyses. Not only would such analyses provide insights into the treatment allocation process with respect to known variables and provide estimates that were standardized to the treated population, but also they could potentially minimize issues of confounding by indication that is common among the psychiatric populations. However, we elected to undertake a matched analysis that was not based on propensity scores matching due to concerns relating to reduction of our sample size and the associated effect on statistical power to detect an association especially given that sudden cardiac death was not a common reported outcome.

## 5. Conclusions

The use of antipsychotic agents in general, whether typical or atypical, was associated with increased risk of all-cause and cardiac mortality. Relative to patients with psychiatric illness who were not exposed to antipsychotic medications olanzapine users demonstrated a significantly increased risk of cardiac mortality, but no increased risk of all-cause mortality. Given concerns regarding the validity of the SCD diagnosis, along with the relatively small number of cases overall, data regarding SCD must be interpreted cautiously.

## Figures and Tables

**Table 1:** Definitions for Outcomes of Interest.

All-cause mortality	Deaths due to any cause except suicide.
Cardiac Mortality	Based on death certificates with ICD 10 codes I10, I11.9, I20, I21, I22, I23, I24, I25, I42.8, I42.9, I46, I47, I49.0, I49.8, I49.9, I51.6, I51.9, I70.9, R09.2, R96, R98.

SCD primary definition	This was a narrow definition of SCD and the most restrictive. The SCD diagnosis was derived from the following ICD10 codes recorded on death certificates (available from January 1998 to November 2010): I46, I47.2, I49.0, R09.2, R96 [10], from Read codes in GPRD that correspond to these ICD10 codes, or from free text in the ± 3 weeks before or after the death recording. Data from a national registry of hospital admission (Hospital Episode Statistics (HES), available from April 1997 to October 2010) were used to ensure that the event took place outside the hospital. A mortality event that occurred within 30 days after the hospital discharge date was not included in the SCD definition.

SCD secondary definition	This definition is broader than the primary definition and is similar to the definition used by Ray et al. (2009) [2]. The SCD diagnosis was derived from death certificates with ICD 10 codes I10, I11.9, I20, I21, I22, I23, I24, I25, I42.8, I42.9, I46, I47, I49.0, I49.8, I49.9, I51.6, I51.9, I70.9, R09.2, R96.1, R98, from Read codes in GPRD that correspond to these ICD10 codes, or from free text in the ± 3 weeks before or after the death recording. Cases with a HES record of hospitalisation were excluded.

SCD tertiary definition	This definition was the same as the secondary definition with the exception that only data from GPRD, and not from death certificates or HES, were used.

SCD (based on free text)	The following text strings were used to identify sudden cardiac death from GPRD free text: “dropped dead”, “died unexpectedly”, “sudden cardiac death”, “death” and “cause unk”, “acute cardiac death”, “unexpected” and “death”, “mors subita”, “death instanta”, “died instanta”, “sudden death”, “dropped death”, and “died suddenly”.

CHD	Based on GPRD Read codes; data from a national registry of hospital admission (Hospital Episode Statistics (HES)) ICD10 codes I21 or I22; or cardiac procedures)

Life-threatening ventricular arrhythmias	Based on GPRD Read codes or HES ICD10 I49.0.

**Table 2:** Baseline characteristics of the patients exposed to antipsychotics and unexposed diseased patients.

Characteristic	Exposed to antipsychotics (*N* = 183,392)	Typical antipsychotics (*N* = 115,491)	Atypical antipsychotics (*N*= 67,901)	Olanzapine (*N* = 20,954)	Psychiatric nonuser (*N* = 193,920)
Prevalent users	67,396 (36.7%)	40,258 (34.9%)	27,138 (40.0%)	9016 (43.0%)	0 (0.0%)
Female	107,226 (58.5%)	69,426 (60.1%)	37,800 (55.7%)	10,296 (49.1%)	132,458 (68.3%)
Male	76,166 (41.5%)	46,065 (39.9%)	30,101 (44.3%)	10,658 (50.9%)	61,462 (31.7%)
Age: mean (sd)	60 (22)	61 (21)	58 (23)	49 (20)	52 (22)
Age: median (IQR)	62 (41–80)	64 (44–80)	58 (38–81)	45 (33–64)	48 (33–75)
Age 18–29	18,474 (10.1%)	9,180 (7.9%)	9,294 (13.7%)	3759 (17.9%)	34,559 (17.8%)
Age 30–39	23,484 (12.8%)	13,747 (11.9%)	9,737 (14.3%)	4237 (20.2%)	38,574 (19.9%)
Age 40–49	23,793 (13.0%)	14,554 (12.6%)	9,239 (13.6%)	3953 (18.9%)	28,555 (14.7%)
Age 50–59	21,244 (11.6%)	14,627 (12.7%)	6,617 (9.7%)	2806 (13.4%)	21,302 (11.0%)
Age 60–69	19,923 (10.9%)	14,461 (12.5%)	5,462 (8.0%)	1,992 (9.5%)	13,831 (7.1%)
Age 70–79	28,088 (15.3%)	18,881 (16.3%)	9,207 (13.6%)	1,948 (9.3%)	21,194 (10.9%)
Age 80+	48,386 (26.4%)	30,041 (26.0%)	18,345 (27.0%)	2,259 (10.8%)	35,905 (18.5%)
BMI: mean (sd)	26 (6)	26 (5)	26 (6)	26 (5)	26 (6)
Nonsmoker	69,482 (37.9%)	44,035 (38.1%)	25,447 (37.5%)	6,771 (32.3%)	80,061 (41.3%)
Exsmoker	29,719 (16.2%)	17,638 (15.3%)	12,081 (17.8%)	2,877 (13.7%)	39,550 (20.4%)
Smoker	51,938 (28.3%)	31,361 (27.2%)	20,577 (30.3%)	8,350 (39.8%)	57,004 (29.4%)
Unknown status	32,253 (17.6%)	22,457 (19.4%)	9,796 (14.4%)	2,956 (14.1%)	17,305 (8.9%)
Hx alcoholism or drug abuse	19,933 (10.9%)	11,429 (9.9%)	8,504 (12.5%)	3,544 (16.9%)	12,572 (6.5%)
Hx suicide attempts	10,777 (5.9%)	5,762 (5.0%)	5,015 (7.4%)	2,015 (9.6%)	8,841 (4.6%)
Schizophrenia	15,475 (8.4%)	6,746 (5.8%)	8,729 (12.9%)	3,750 (17.9%)	7,779 (4.0%)
Bipolar/other mood disorders	7,368 (4.0%)	3,170 (2.7%)	4,198 (6.2%)	2,008 (9.6%)	4,700 (2.4%)
Major depression	19,981 (10.9%)	11,680 (10.1%)	8,301 (12.2%)	3,343 (16.0%)	134,105 (69.2%)
Dementia	25,174 (13.7%)	12,669 (11.0%)	12,505 (18.4%)	1,291 (6.2%)	47,336 (24.4%)
No recorded psychiatric disorders	115,394 (62.9%)	81,226 (70.3%)	34,168 (50.3%)	10,562 (50.4%)	0 (0.0%)
Diabetes	15,276 (8.3%)	9,480 (8.2%)	5,796 (8.5%)	1,047 (5.0%)	13,639 (7.0%)
Acute MI	6,530 (3.6%)	4,224 (3.7%)	2,306 (3.4%)	380 (1.8%)	5,466 (2.8%)
Statins/fibrates in previous 3 mo.	16,203 (8.8%)	8,406 (7.3%)	7,797 (11.5%)	1,464 (7.0%)	18,124 (9.3%)
Antiplatelets	32,260 (17.6%)	18,900 (16.4%)	13,360 (19.7%)	2,096 (10.0%)	25,397 (13.1%)
Antidepressants	83,595 (45.6%)	49,545 (42.9%)	34,050 (50.1%)	11,487 (54.8%)	126,688 (65.3%)
Anxiolytics	27,389 (14.9%)	15,637 (13.5%)	11,752 (17.3%)	3,898 (18.6%)	12,907 (6.7%)
SSRIs	44,428 (24.2%)	24,330 (21.1%)	20,098 (29.6%)	6,757 (32.2%)	98,373 (50.7%)
Lithium	5,122 (2.8%)	2,851 (2.5%)	2,271 (3.3%)	1,053 (5.0%)	1,743 (0.9%)
Antiepileptics	10,090 (5.5%)	5,853 (5.1%)	4,237 (6.2%)	1,287 (6.1%)	3,817 (2.0%)

**Table 3:** Relative risks of all-cause mortality (excluding suicide) and cardiovascular outcomes for current users of various types of antipsychotics versus nonuser psychiatric controls.

Outcome	Exposure type	Number of events	PY	Incidence rate per 1000 PY (95% CI)	Age-and sex-adjusted relative risk (RR) (95% CI)	Fully adjusted RR (aRR) (95% CI)
All-cause mortality	Nonuser psychiatric	7765	307,276	25.3 (24.7–25.8)	Reference	Reference
	Atypical or typical	23,841	276,892	86.1 (85.0–87.2)	2.15 (2.10–2.21)^‡^	1.75 (1.64–1.87)^‡^
	Atypical	7356	94,112	78.2 (76.4–80.0)	1.76 (1.71–1.82)^‡^	1.52 (1.40–1.64)^‡^
	Typical	15473	167,005	92.7 (91.2–94.1)	2.34 (2.28–2.41)^‡^	1.82 (1.69–1.98)^‡^
	Olanzapine	1305	34,985	37.3 (35.3–39.4)	1.40 (1.32–1.48)^‡^	1.04 (0.93–1.17)

Cardiac mortality	Nonuser psychiatric	1289	200,988	6.4 (6.1–6.8)	Reference	Reference
	Atypical or typical	2478	145,643	17.0 (16.4–17.7)	1.62 (1.52–1.74)^‡^	1.72 (1.42–2.07)^‡^
	Atypical	1200	73,284	16.4 (15.5–17.3)	1.47 (1.36–1.59)^‡^	1.74 (1.40–2.14)^‡^
	Typical	1180	64,913	18.2 (17.2–19.3)	1.78 (1.64–1.92)^‡^	1.88 (1.49–2.39)^‡^
	Olanzapine	206	26,765	7.7 (6.7–8.8)	1.25 (1.07–1.44)^‡^	1.53 (1.12–2.09)^‡^

SCD—primary definition	Nonuser psychiatric	10	23,872	0.4 (0.2–0.8)	Reference	Reference
	Atypical or typical	44	16,144	2.7 (2.0–3.7)	5.76 (2.90–11.45)^‡^	5.76 (2.90–11.45)^‡^
	Atypical	21	10,201	2.1 (1.3–3.2)	0 (NC)	0 (NC)
	Typical	21	5415	3.9 (2.4–5.9)	0 (NC)	0 (NC)
	Olanzapine	6	3454	1.7 (0.6–3.8)	0 (NC)	0 (NC)

SCD—secondary definition	Nonuser psychiatric	98	23,873	4.1 (3.3–5.0)	Reference	Reference
	Atypical or typical	178	16,144	11.0 (9.5–12.8)	2.36 (1.84–3.02)^‡^	2.15 (1.64–2.81)^‡^
	Atypical	90	10,201	8.8 (7.1–10.8)	1.93 (1.45–2.57)^‡^	1.79 (1.33–2.42)^‡^
	Typical	78	5415	14.4 (11.4–18.0)	2.82 (2.09–3.80)^‡^	3.99 (1.29–12.40)^†^
	Olanzapine	10	3454	2.9 (1.4–5.3)	1.33 (0.69– 2.57)	0 (NC)

SCD—tertiary definition	Nonuser psychiatric	119	64,880	1.8 (1.5–2.2)	Reference	Reference
	Atypical or typical	198	43,550	4.6 (3.9–5.2)	1.92 (1.53–2.42)^‡^	1.79 (1.42–2.27)^‡^
	Atypical	97	26,345	3.7 (3.0–4.5)	1.60 (1.22–2.09)^‡^	1.51 (1.14–1.99)^‡^
	Typical	96	15,621	6.2 (5.0–7.5)	2.39 (1.82–3.13)^‡^	2.27 (1.71–3.01)^‡^
	Olanzapine	15	8425	1.8 (1.0–2.9)	1.61 (0.93–2.77)*	0 (NC)

CHD	Nonuser psychiatric	982	278,330	3.5 (3.3–3.8)	Reference	Reference
	Atypical or typical	1355	236,229	5.7 (5.4–6.1)	1.08 (1.00–1.18)*	1.16 (0.94–1.44)
	Atypical	444	86,115	5.2 (4.7–5.7)	0.94 (0.84–1.05)	0.98 (0.76–1.26)
	Typical	873	136,777	6.4 (6.0–6.8)	1.19 (1.09–1.31)^‡^	1.39 (1.09–1.76)^‡^
	Olanzapine	132	31,806	4.2 (3.5–4.9)	1.01 (0.84–1.21)	1.07 (0.76–1.49)

Ventricular arrhythmias	Nonuser psychiatric	500	279,243	1.8 (1.6–2.0)	Reference	Reference
	Atypical or typical	646	237,647	2.7 (2.5–2.9)	1.14 (1.01–1.28)^†^	1.16 (1.02–1.31)^†^
	Atypical	230	86,319	2.7 (2.3–3.0)	1.05 (0.89–1.23)	1.06 (0.90–1.26)
	Typical	382	137,982	2.8 (2.5–3.1)	1.16 (1.01–1.32)^†^	1.37 (1.00–1.87)*
	Olanzapine	55	31,921	1.7 (1.3–2.2)	0.90 (0.68–1.18)	0.86 (0.65–1.14)

*P < 0.10, ^†^ *P* < 0.05, ^‡^ *P* < 0.01.

Abbreviations: aRR: adjusted relative risk; CHD: coronary heart disease; CI: confidence interval; NC: not calculated; PY: person-years; SCD: sudden cardiac death.

**Table 4:** All-cause mortality (excluding suicide), cardiac mortality, and sudden cardiac death: current antipsychotic use versus nonuser psychiatric.

Outcome	Treatment	Number of events	PY	Incidence rate per 1000 PY (95% CI)	Fully adjusted RR (aRR) (95% CI)
All-cause mortality	Nonuser psychiatric	7765	307,276	25.3 (24.7–25.8)	Reference
	Amisulpride	503	5220	96.4 (88.1–105.2)	1.51 (1.37–1.66)^‡^
	Chlorpromazine	1553	28,550	54.4 (51.7–57.2)	1.59 (1.49–1.69)^‡^
	Haloperidol	5552	21,045	263.8 (256.9–270.9)	2.33 (2.12–2.56)^‡^
	Olanzapine	1305	34,985	37.3 (35.3–39.4)	1.04 (0.93–1.17)
	Perphenazine	260	8507	30.6 (27.0–34.5)	1.30 (0.91–1.85)
	Quetiapine	1887	17,216	109.6 (104.7–114.7)	1.42 (1.34–1.50)^‡^
	Risperidone	3600	33,917	106.1 (102.7–109.7)	1.64 (1.56–1.72)^‡^

Cardiac mortality	Nonuser psychiatric	1289	200,988	6.4 (6.1–6.8)	Reference
	Amisulpride	98	3936	24.9 (20.2–30.3)	2.50 (1.76–3.57 )^‡^
	Chlorpromazine	161	15,220	10.6 (9.0–12.3)	1.70 (1.11–2.58) )^†^
	Haloperidol	522	11,546	45.2 (41.4–49.3)	2.10 (1.59–2.77) ^‡^
	Olanzapine	206	26,765	7.7 (6.7–8.8)	1.53 (1.12– 2.09) ^‡^
	Perphenazine	22	3232	6.8 (4.3–10.3)	0 (NC)
	Quetiapine	344	15,183	22.7 (20.3–25.2)	1.79 (1.38–2.33)^ ‡^
	Risperidone	543	25,024	21.7 (19.9–23.6)	1.87 (1.44 –2.41)^‡^

Sudden cardiac death (secondary definition)	Nonuser psychiatric	98	23,873	4.1 (3.3–5.0)	Reference
	Amisulpride	15	674	22.3 (12.5–36.7)	0 (NC)
	Chlorpromazine	10	1297	7.7 (3.7–14.2)	0 (NC)
	Haloperidol	47	1236	38.0 (28.0–50.6)	4.33 (3.03–6.17)^‡^
	Olanzapine	10	3454	2.9 (1.4–5.3)	0 (NC)
	Perphenazine	0	221	0.0 (0.0–16.7)	0 (NC)
	Quetiapine	46	3407	13.5 (9.9–18.0)	1.51 (1.04–2.19)^†^
	Risperidone	17	2187	7.8 (4.5–12.4)	0 (NC)

*P < 0.10; ^†^ *P* < 0.05; ^‡^ *P* < 0.01.

Abbreviations: aRR: adjusted relative risk; CI: confidence interval; NC: not calculated; PY: person-years.

**Table 5:** Relative risks of all-cause mortality (excluding suicide) and cardiovascular outcomes for current users of various types of antipsychotics versus nonuser psychiatric controls stratified by dose.

Outcome	Exposure type of current users	Dose	Outcome events	Person-time (Years)	Incidence rate per 1000 PY (95% CI)	Fully adjusted RR (aRR) (95% CI)
All-cause mortality	Nonuser psychiatric	NA	7765	307,276	25.3 (24.7–25.8)	Reference
	Atypical or typical	Low	21,208	218,635	97.0 (95.7–98.3)	1.72 (1.62–1.84)^‡^
		Medium	1978	37,704	52.5 (50.2–54.8)	1.94 (1.80–2.10)^‡^
		High	655	20,553	31.9 (29.5–34.4)	1.89 (1.71–2.09)^‡^
	Atypical	Low	5969	51,838	115.2 (112.3–118.1)	1.51 (1.40–1.64)^‡^
		Medium	1106	28,874	38.3 (36.1–40.6)	1.54 (1.40–1.69)^‡^
		High	281	13,401	21.0 (18.6–23.6)	1.48 (1.29–1.71)^‡^
	Typical	Low	14,528	160,264	90.7 (89.2–92.1)	1.76 (1.63–1.89)^‡^
		Medium	683	4232	161.4 (149.5–174.0)	3.74 (3.36–4.15)^‡^
		High	262	2509	104.4 (92.2–117.9)	3.61 (3.14–4.16)^‡^
	Olanzapine	Low	873	17,172	50.8 (47.5–54.3)	1.00 (0.88–1.13)
		Medium	323	12,304	26.3 (23.5–29.3)	1.07 (0.92–1.24)
		High	109	5509	19.8 (16.3–23.9)	1.20 (0.97–1.49)*

Cardiac Mortality	Nonuser psychiatric	NA	1289	200,988	6.4 (6.1–6.8)	Reference
	Atypical or typical	Low	2157	107,874	20.0 (19.2–20.9)	1.65 (1.36–2.00)^‡^
		Medium	251	25,299	9.9 (8.7–11.2)	2.10 (1.68–2.63)^‡^
		High	70	12,470	5.6 (4.4–7.1)	2.27 (1.68–3.07)^‡^
	Atypical	Low	984	42,824	23.0 (21.6–24.5)	1.35 (1.24–1.47)^‡^
		Medium	173	21,258	8.1 (7.0–9.5)	1.67 (1.42–1.97)^‡^
		High	43	9202	4.7 (3.4–6.3)	1.84 (1.35–2.51)^‡^
	Typical	Low	1108	61,999	17.9 (16.8–19.0)	1.82 (1.44–2.32)^‡^
		Medium	57	1862	30.6 (23.2–39.7)	3.40 (2.39–4.83)^‡^
		High	15	1053	14.3 (8.0–23.5)	3.13 (1.79–5.47)^‡^
	Olanzapine	Low	129	13,897	9.3 (7.8–11.0)	1.31 (0.93–1.82)
		Medium	60	8851	6.8 (5.2–8.7)	2.06 (1.41–3.01)^‡^
		High	17	4016	4.2 (2.5–6.8)	2.37 (1.36–4.12)^‡^

SCD (secondary definition)	Nonuser psychiatric	NA	98	23,873	4.1 (3.3–5.0)	Reference
	Atypical or typical	Low	157	11,914	13.2 (11.2–15.4)	2.09 (1.60–2.75)^‡^
		Medium	14	2956	4.7 (2.6–8.0)	2.42 (1.35–4.35)^‡^
		High	7	1274	5.5 (2.2–11.3)	3.36 (1.50–7.50)^‡^
	Atypical	Low	82	6469	12.7 (10.1-15.7)	1.78 (1.31–2.42)^‡^
		Medium	6	2660	2.3 (0.8–4.9)	1.32 (0.57–3.05)
		High	2	1072	1.9 (0.2–6.7)	1.53 (0.37–6.39)
	Typical	Low	70	5202	13.5 (10.5–17.0)	2.48 (1.81–3.40)^‡^
		Medium	5	156	32.1 (10.4–74.8)	6.07 (2.45–14.99)^‡^
		High	3	57	52.8 (10.9–154.3)	10.05 (3.15–32.08)^‡^
	Olanzapine	Low	8	1951	4.1 (1.8–8.1)	0 (NC)
		Medium	2	1052	1.9 (0.2–6.9)	0 (NC)
		High	0	451	0.0 (0.0-8.2)	0 (NC)

CHD	Nonuser psychiatric	NA	982	278,330	3.5 (3.3–3.8)	Reference
	Atypical or typical	Low	1192	185,032	6.4 (6.1–6.8)	1.19 (0.96–1.48)
		Medium	126	33,440	3.8 (3.1–4.5)	1.15 (0.88–1.50)
		High	37	17,757	2.1 (1.5 –2.9)	0.86 (0.59–1.26)
	Atypical	Low	328	48,223	6.8 (6.1–7.6)	0.94 (0.72–1.23)
		Medium	95	26,059	3.7 (3.0–4.5)	1.11 (0.81–1.51)
		High	21	11,833	1.8 (1.1–2.7)	0.77 (0.47–1.26)
	Typical	Low	843	131,363	6.4 (6.0–6.9)	1.40 (1.10–1.78)^‡^
		Medium	20	3413	5.9 (3.6–9.1)	1.25 (0.76–2.05)
		High	10	2001	5.0 (2.4–9.2)	1.54 (0.80–2.98)
	Olanzapine	Low	76	15,856	4.8 (3.8–6.0)	0.99 (0.68–1.45)
		Medium	46	11,029	4.2 (3.1–5.6)	1.34 (0.89–2.01)
		High	10	4921	2.0 (1.0–3.7)	0.82 (0.42–1.63)

Ventricular arrhythmias	Nonuser psychiatric	NA	500	279,243	1.8 (1.6–2.0)	Reference
	Atypical or typical	Low	540	186,417	2.9 (2.7–3.2)	1.13 (0.99–1.29)*
		Medium	70	33,445	2.1 (1.6–2.6)	1.27 (0.98–1.64)*
		High	36	17,784	2.0 (1.4–2.8)	1.55 (1.08–2.20)^†^
	Atypical	Low	163	48,398	3.4 (2.9–3.9)	1.03 (0.85–1.24)
		Medium	48	26,079	1.8 (1.4–2.4)	1.12 (0.83–1.51)
		High	19	11,842	1.6 (1.0–2.5)	1.27 (0.79–2.02)
	Typical	Low	359	132,550	2.7 (2.4–3.0)	1.13 (0.98–1.31)*
		Medium	16	3416	4.7 (2.7–7.6)	2.23 (1.35–3.69)^‡^
		High	7	2017	3.5 (1.4–7.2)	2.11 (0.99–4.47)*
	Olanzapine	Low	33	15,926	2.1 (1.4–2.9)	0.84 (0.59–1.20)
		Medium	14	11,068	1.3 (0.7–2.1)	0.78 (0.46–1.33)
		High	8	4927	1.6 (0.7–3.2)	1.22 (0.60–2.46)

*P < 0.10, ^†^ *P* < 0.05, ^‡^ *P* < 0.01.

Abbreviations: aRR: adjusted relative risk; CHD: coronary heart disease; CI: confidence interval; NC: not calculable; PY: person-years; SCD: sudden cardiac death.

**Table 6:** Relative risks of all-cause mortality (excluding suicide) and cardiac mortality for current users of various types of antipsychotics versus nonuser psychiatric controls stratified by duration of treatment.

Outcome	Exposure type of current users	Duration of treatment	Outcome events	Person-time (Years)	Incidence rate per 1000 PY (95% CI)	Fully adjusted RR (aRR) (95% CI)
All-cause mortality	Nonuser psychiatric	NA	7765	307,276	25.3 (24.7–25.8)	Reference
	Atypical or typical	<1 year	16,571	176,402	93.9 (92.5–95.4)	1.95 (1.83–2.08)^‡^
		1–3 years	4861	56,041	86.7 (84.3–89.2)	1.53 (1.43–1.64)^‡^
		>3 years	2409	44,449	54.2 (52.1–56.4)	1.20 (1.11–1.29)^‡^
	Atypical	<1 year	4333	49,387	87.7 (85.1–90.4)	1.68 (1.55–1.82)^‡^
		1–3 years	2068	26407	78.3 (75.0–81.8)	1.42 (1.31–1.55)^‡^
		>3 years	955	18,318	52.1 (48.9–55.6)	1.15 (1.04–1.27)^‡^
	Typical	<1 year	11,989	124,518	96.3 (94.6–98.0)	1.98 (1.84–2.13)^‡^
		1–3 years	2430	24,906	97.6 (93.7–101.5)	1.56 (1.43–1.69)^‡^
		>3 years	1054	17,581	60.0 (56.4–63.7)	1.17 (1.06–1.28)^‡^
	Olanzapine	<1 year	659	18,111	36.4 (33.7–39.3)	1.21 (1.06–1.38)^‡^
		1–3 years	395	9674	40.8 (36.9–45.1)	1.04 (0.91–1.20)
		>3 years	251	7200	34.9 (30.7–39.5)	0.78 (0.67–0.91)^‡^

Cardiac mortality	Nonuser psychiatric	NA	1289	200,988	6.4 (6.1–6.8)	Reference
	Atypical or typical	<1 year	1707	95,129	17.9 (17.1–18.8)	1.85 (1.53–2.24)^‡^
		1–3 years	563	32,383	17.4 (16.0–18.9)	1.60 (1.30–1.95)^‡^
		>3 years	208	18,131	11.5 (10.0–13.1)	1.31 (1.04–1.65)^†^
	Atypical	<1 year	761	42,705	17.8 (16.6–19.1)	1.51 (1.38–1.66)^‡^
		1–3 years	315	20,399	15.4 (13.8–17.2)	1.26 (1.11–1.43)^‡^
		>3 years	124	10,180	12.2 (10.1–14.5)	1.16 (0.97–1.41)
	Typical	<1 year	918	50,850	18.1 (16.9–19.3)	1.92 (1.51–2.45)^‡^
		1–3 years	204	9216	22.1 (19.2–25.4)	1.75 (1.34–2.28)^‡^
		>3 years	58	4848	12.0 (9.1–15.5)	1.21 (0.86–1.71)
	Olanzapine	<1 year	111	15,615	7.1 (5.9–8.6)	1.67 (1.18–2.35)‡
		1–3 years	65	7269	8.9 (6.9–11.4)	1.57 (1.09–2.26)^†^
		>3 years	30	3881	7.7 (5.2–11.0)	1.24 (0.79–1.94)

*P < 0.10, ^†^ *P* < 0.05, ^‡^ *P* < 0.01.

Abbreviations: aRR: adjusted relative risk; CI: confidence interval; NC: not calculable; PY: person-years.

## References

[1] Haddad PM, Sharma SG (2007). Adverse effects of atypical antipsychotics: differential risk and clinical implications. *CNS Drugs*.

[2] Ray WA, Chung CP, Murray KT, Hall K, Stein CM (2009). Atypical antipsychotic drugs and the risk of sudden cardiac death. *The New England Journal of Medicine*.

[3] Ray WA, Meredith S, Thapa PB, Meador KG, Hall K, Murray KT (2001). Antipsychotics and the risk of sudden cardiac death. *Archives of General Psychiatry*.

[4] Schneeweiss S, Setoguchi S, Brookhart A, Dormuth C, Wang PS (2007). Risk of death associated with the use of conventional versus atypical antipsychotic drugs among elderly patients. *Canadian Medical Association Journal*.

[5] Enger C, Weatherby L, Reynolds RF, Glasser DB, Walker AM (2004). Serious cardiovascular events and mortality among patients with schizophrenia. *Journal of Nervous and Mental Disease*.

[6] Gardner D, Murphy A, ’Donnell H O, Centorrino F, Baldessarini R (2010). International consensus study of antipsychotic dosing. *The American Journal of Psychiatry*.

[7] Hosmer DW, Lemeshow S (2000). *Applied Logistic Regression*.

[8] Vittinghoff E, McCullogh CE (2007). Relaxing the rule of ten events per variable in logistic and cox regression. *American Journal of Epidemiology*.

[9] Ramlau-Hansen H (1983). Smoothing counting process intensities by means of kernel functional. *Annals of Statistics*.

[10] Hennessy S, Leonard CE, Freeman CP (2010). Validation of diagnostic codes for outpatient-originating sudden cardiac death and ventricular arrhythmia in Medicaid and Medicare claims data. *Pharmacoepidemiology and Drug Safety*.

[11] Curkendall SM, Mo J, Glasser DB, Stang MR, Jones JK (2004). Cardiovascular disease in patients with schizophrenia in Saskatchewan, Canada. *Journal of Clinical Psychiatry*.

[12] Goff DC, Sullivan LM, McEvoy JP (2005). A comparison of ten-year cardiac risk estimates in schizophrenia patients from the CATIE study and matched controls. *Schizophrenia Research*.

[13] Hennessy S, Leonard CE, Palumbo CM, Bilker WB, Newcomb C, Kimmel SE (2008). Diagnostic codes for sudden cardiac death and ventricular arrhythmia functioned poorly to identify outpatient events in EPIC’s General Practice Research Database. *Pharmacoepidemiology and Drug Safety*.

[14] Osborn DPJ, Levy G, Nazareth I, Petersen I, Islam A, King MB (2007). Relative risk of cardiovascular and cancer mortality in people with severe mental illness from the United Kingdom’s General Practice Research Database. *Archives of General Psychiatry*.

[15] Tiihonen J, Lönnqvist J, Wahlbeck K (2009). 11-year follow-up of mortality in patients with schizophrenia: a population-based cohort study (FIN11 study). *The Lancet*.

[16] Leonard CE, Freeman CP, Newcomb CW (2013). Antipsychotics and the risks of sudden cardiac death and all-cause death: cohort studies in Medicaid and dually-eligible Medicaid-Medicare beneficiaries of five states. *Journal of Clinical & Experimental Cardiology*.

[17] Taylor DM (2003). Antipsychotics and QT prolongation. *Acta Psychiatrica Scandinavica*.

[18] Czekalla J, Beasley C.M. J, Dellva MA, Berg PH, Grundy S (2001). Analysis of the QTc interval during olanzapine treatment of patients with schizophrenia and related psychosis. *Journal of Clinical Psychiatry*.

[19] Czekalla J, Kollack-Walker S, Beasley C.M. J (2001). Cardiac safety parameters of olanzapine: comparison with other atypical and typical antipsychotics. *Journal of Clinical Psychiatry*.

[20] Dixon L, Postrado L, Delahanty J, Fischer PJ, Lehman A (1999). The association of medical comorbidity in schizophrenia with poor physical and mental health. *Journal of Nervous and Mental Disease*.

[21] Barth J, Schumacher M, Herrmann-Lingen C (2004). Depression as a risk factor for mortality in patients with coronary heart disease: a meta-analysis. *Psychosomatic Medicine*.

[22] Priori SG, Aliot E, Blomstrom-Lundqvist C (2001). Task force on sudden cardiac death of the European society of cardiology. *European Heart Journal*.

